# Viral Vaccines as an Alternative to Antimicrobials: A Perspective from Swine Veterinarians on Challenges, Opportunities, and Future Directions

**DOI:** 10.3390/pathogens14121259

**Published:** 2025-12-09

**Authors:** Danqin Li, Xirui Zhang, Michael D. Apley, Jordan T. Gebhardt, Locke Karriker, Joseph F. Connor, Corinne Bromfield, Brian Lubbers, Hatem Kittana, Dustin Pendell, Rachel Madera, Nina Muro, Aidan Craig, Brooke Shenkenberg, Yuzhen Li, Lihua Wang, Jishu Shi

**Affiliations:** 1 Center on Biologics Development and Evaluation, College of Veterinary Medicine, Kansas State University, Manhattan, KS 66506, USA; danqinli@vet.k-state.edu (D.L.); xzr92@missouri.edu (X.Z.); rachelmadera@vet.k-state.edu (R.M.); nina14@vet.k-state.edu (N.M.); aidan3@vet.k-state.edu (A.C.); shenky@vet.k-state.edu (B.S.); yuzhen@vet.k-state.edu (Y.L.); 2 Department of Anatomy and Physiology, College of Veterinary Medicine, Kansas State University, Manhattan, KS 66506, USA; mapley@vet.k-state.edu; 3 College of Veterinary Medicine, University of Missouri, Columbia, MO 65211, USA; bromfieldc@missouri.edu; 4 Department of Diagnostic Medicine/Pathobiology, College of Veterinary Medicine, Kansas State University, Manhattan, KS 66506, USA; jgebhardt@vet.k-state.edu (J.T.G.); hkittana@vet.k-state.edu (H.K.); 5 Department of Veterinary Diagnostic and Production Animal Medicine, College of Veterinary Medicine, Iowa State University, Ames, IA 50011, USA; karriker@iastate.edu; 6 Carthage Veterinary Service, Ltd., Carthage, IL 62321, USA; connor@hogvet.com; 7 Department of Clinical Sciences, College of Veterinary Medicine, Kansas State University, Manhattan, KS 66506, USA; blubbers@vet.k-state.edu; 8 Department of Agricultural Economics, College of Agriculture, Kansas State University, Manhattan, KS 66506, USA; dpendell@k-state.edu

**Keywords:** antimicrobial resistance, alternatives, viral vaccines, swine veterinarian, challenges, opportunities

## Abstract

Antimicrobial resistance (AMR) is an increasing concern in food animal production. In swine herds, viral infections often lead to secondary bacterial disease and higher antimicrobial use (AMU). This study describes how U.S. swine veterinarians view the role of viral vaccines in reducing this reliance on antimicrobials. We conducted a national survey of swine practitioners and follow-up semi-structured interviews with a subset of respondents. Across participants, porcine reproductive and respiratory syndrome (PRRS), swine influenza (SIV), and rotaviral enteritis were most often named as viral diseases in urgent need of improved vaccines. These diseases cause substantial economic losses and frequently trigger AMU in commercial herds. Veterinarians reported several recurring challenges with current vaccines, including limited cross-protection against field strains, interference from maternally derived antibodies, and short duration of protection. Despite these limitations, most respondents supported vaccination as a key tool to curb AMU and indicated they would accept higher prices for clearly improved products. These findings reveal both a clear need and specific opportunities for future vaccine development to provide broader and more reliable protection, reduce AMU, and help slow the development of AMR.

## 1. Introduction

The use of antimicrobials in food animal production has a history of over 80 years, with the first use dating back to the 1940s [1]. Antimicrobials have played a significant role in enhancing animal productivity and meeting the growing food demand of a rapidly increasing global population [2]. However, their overuse has created a major global health crisis: antimicrobial resistance (AMR). Within the “One Health” framework, AMR not only limits treatment options for veterinarians and compromises animal health, but also poses a direct threat to human health through the food chain. Research suggests that a 10% increase in animal antimicrobial use can lead to a 0.3% increase in human AMR [3]. Therefore, curbing antimicrobial use (AMU) in food animals is a crucial step toward protecting both animal and human health.

To address these concerns, the U.S. Food and Drug Administration (FDA) has implemented various policies aimed at regulating antimicrobial use in food animals. Notably, Guidance for Industry (GFI) #213, which took effect on 1 January 2017, prohibited the use of medically important antimicrobials for growth promotion purposes [4]. This guidance initially led to a significant decline in total antimicrobial sales, but the downward trend began to slowdown in 2018. Within food animal production, antimicrobial use in the swine industry has raised the greatest public concern. According to the FDA, in 2023 swine accounted for the highest percentage (44%) of medically important antimicrobial sales among all food-producing animals [5]. This concerning trend underscores the urgent need to promote the judicious use of antimicrobials in the swine industry to mitigate the risk of AMR.

A significant driver of AMU in swine is the use of antimicrobials to treat secondary bacterial infections that often follow viral diseases. Viruses can damage the respiratory and intestinal barriers, making pigs more susceptible to bacterial infections. Studies have shown that in porcine reproductive and respiratory syndrome (PRRS)-positive herds, antimicrobial use increases by 379% in nursery pigs and 274% in pigs approaching market weight [6]. Vaccines offer a promising solution by reducing the burden of viral diseases and, in turn, the reliance on antimicrobials. An ideal vaccine should be effective, safe, affordable, and readily available, but many existing vaccines fall short of these expectations. Given their crucial role in advising producers, understanding veterinarians’ perspectives on the challenges and opportunities of current viral vaccines is essential. This study aims to address that gap by gathering insights from U.S. swine veterinarians. We explore their perspectives on the prioritization of swine diseases and on the limitations of existing viral vaccines, including concerns about safety, efficacy, availability, and cost, to identify opportunities for improvement. This information can help guide the development of improved vaccines that protect animal health, reduce AMU in the swine industry, and help mitigate the global threat of antimicrobial resistance.

## 2. Materials and Methods

### 2.1. Ethics Statement

The study materials and research protocol were approved by the Kansas State University Research Compliance Office (Protocol # IRB-12219) prior to project started. All participants were requested to sign consent at the beginning of the survey, and anonymity was assured throughout. Participants had the option to provide their email address voluntarily at the end of the survey to receive a $25 gift card as a token of appreciation.

### 2.2. Survey Design and Dissemination

This study consists of two phases to collect both quantitative and qualitative data. In the first phase, an online survey was distributed to collect quantitative data. At the conclusion of the survey, participants were asked if they would be willing to participate in a follow-up interview. In the second phase, participants who expressed interest were invited to one-on-one semi-structured interviews to gather qualitative insights.

The swine veterinarians practiced in the United States were the target population for this survey. Our survey was implemented using the Qualtrics software (Version: 08/10/2024), Copyright © 2024 Qualtrics, Provo, UT, USA. Available at http://www.qualtrics.com (accessed on 10 August 2024), access with license through the Kansas State University (KSU), KS, USA. The survey invitations to participate were distributed via personalized emails to American Association of Swine Veterinarians (AASV) membership lists (n = 200) between June and July 2024. Although this may introduce some selection bias by potentially underrepresenting veterinarians who work with swine but are not AASV members, the AASV nonetheless represents the majority of U.S. swine practitioners. A reminder email was sent one week after the initial invitation. The survey remained open for a total of eight weeks, concluding in August 2024.

The questionnaire was designed based on insights from the literature and the expertise of authors specializing in swine veterinary medicine and agricultural economics. The questionnaire used the branching logic format, allowing the survey adapted dynamically to participants’ responses by skipping irrelevant questions (e.g., if the participants did not identify Porcine Reproductive and Respiratory Syndrome (PRRS) as a swine disease that urgently need a better vaccine, they will not receive the following sub-questions designed for PRRS). Therefore, individual participants answered a variable number of questions. On average, the survey took 40 min to complete (after excluding one outlier caused by prolonged survey inactivity.) Because of the branching logic, the total number of questions varied among respondents. Participants who completed at least 80% of the survey typically selected an average of eight diseases (two systemic, three enteric, and three respiratory), and each selected disease generated approximately 20 follow-up questions. As a result, fully eligible participants generally answered between 128 and 160 questions in total, depending on their specific survey pathway.

**Figure 1:**
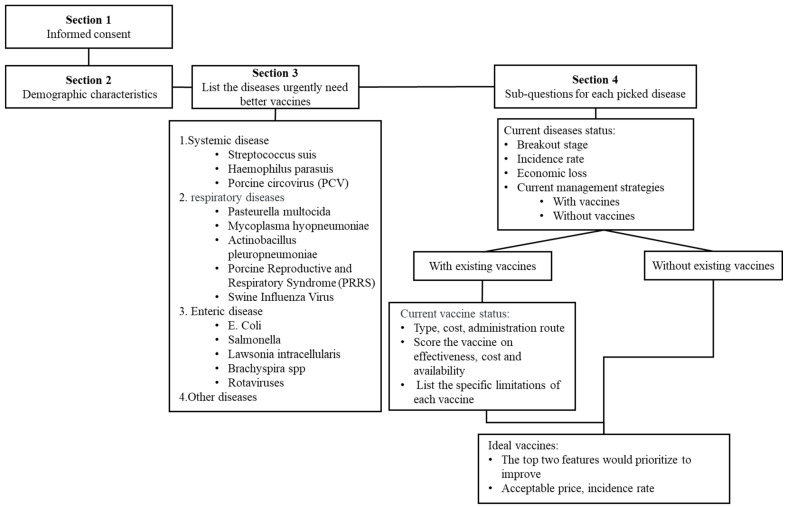
The workflow of the survey.

The survey consisted of four main sections (Figure 1): (i) Informed consent: Participants were required to read and agree to the informed consent form before proceeding. (ii) Demographic characteristics and general practice information: This section included three questions on years of practice, the number of pigs served, and the geographic region of practice. (iii) Swine diseases in urgent need of improved vaccines: This section included four questions focusing on systemic, respiratory, gastrointestinal, and other diseases categories, covering a total of 13 common swine diseases. (iv) Detailed question for each selected disease: For each disease selected by participants, 32 structured questions were presented covering the following areas: (a) Current disease status (e.g., production stages affected, incidence rates, and economic impact, etc.). (b) Current management strategies (based on participants answers, distinguishing disease into with existing vaccines and those without, two categories). (c) For the diseases with existing vaccines, participants graded the vaccine on three aspects including efficacy, availability, and cost, on a scale of 1 to 5 (where 1 indicated “not ideal” and 5 indicated “extremely ideal”). Participants identified specific limitations contributing to the challenges of the current vaccine on adverse effects and the aspects that graded lower than five. Then identified limitations formed a pool of current vaccine challenges. Participants selected the top two challenges from the pool that they believed should be prioritized for improvement. Based on these improvements, participants were also asked to indicate the desired incidence rate and the acceptable cost increase for the improved vaccine. (d) For the disease without existing vaccine, participants identified the top two features they desired in a future vaccine. Similarly, participants were asked to indicate the desired incidence rate and the acceptable cost for the future vaccine.

### 2.3. Interview

Semi-structured interviews were tailored for each participant based on their survey responses. Between June and July 2024, five swine veterinarians participated in interviews, four conducted via Zoom and one in person. All interviews were hosted and documented by the first author. The interviews were all audio recorded with participants’ consent. Recordings were used only to ensure the accuracy of notes, and access to recordings was restricted to the first author.

Each interview lasted approximately 40 min with an average of eight open-ended questions. The interview focused on following 7 key areas: (i) The relationship between vaccines and antibiotics in swine health management; (ii) Priority diseases for vaccine development and disease control strategies; (iii) Economic considerations of vaccine adoption; (iv) Regulatory, educational, and logistical challenges in vaccine implementation; (v) Prescription platform vaccines and custom vaccine development; (vi) Future trends in vaccine development and antimicrobial use reduction; and (vii) Challenges in disease surveillance and data collection. These interviews aimed to further understand U.S. swine veterinarians’ awareness and attitudes toward reducing antimicrobials use through vaccine adoption.

### 2.4. Data and Statistical Analysis

For the survey data, we first exported results from Qualtrics into Microsoft Excel (Version 2509 Build 16.0.19231.20138) for initial organization and summarization. Subsequently, these data were imported into GraphPad Prism (version 10) for further statistical analysis and visualization. The figure “Regional distribution of all respondents” was generated by Power BI (Version 2.131.901.0), while the rest of the figures were visualized using GraphPad Prism 10. We used a two-way ANOVA with Bonferroni correction to compare morbidity and mortality rates across vaccine type (current vs. ideal) and stage (pre-wean, nursery, grower/finisher), even with relatively small sample sizes, to examine the main effects. While we acknowledge that small sample sizes can reduce statistical power, ANOVA remains appropriate for identifying patterns across multiple factors when assumptions are reasonably met. The comparison of current and ideal vaccine prices was performed using a two-tailed, unpaired t-test with Welch’s correction, which is more reliable than the standard *t*-test for small groups. All figures use a 95% confidence interval to measure the significance between groups. All significant results (*p*-value < 0.05) were indicated with asterisks in the figures.

For interviews, recordings were transcribed verbatim and anonymized. The transcripts were reviewed multiple times to ensure accuracy and familiarity with the content. Key points from the interviews were grouped into major themes that consistently appeared across multiple participants. These themes encompassed shared perspectives on vaccine effectiveness, economic factors, regulatory and logistical challenges, and future vaccine development priorities. Responses were also compared to identify both common viewpoints and individual differences. To provide a more comprehensive understanding of swine veterinarians’ perspectives on using vaccines as an alternative to antimicrobials, we integrated our interview findings with the survey data.

A five-point Numeric Rating Scale (NRS) was adopted to assess multiple dimensions of vaccine evaluation, including effectiveness, cost, and availability. For effectiveness, a score of 1 indicated not effective at all and 5 indicated extremely effective. For cost, a score of 1 represented very cheap and 5 represented very expensive. For availability, a score of 1 indicated extremely difficult to obtain and 5 indicated extremely easy to obtain. To minimize potential misunderstanding of the scale, each numeric option was accompanied by descriptive text in the questionnaire. Responses were treated as ordinal data but summarized as mean values with 95% confidence intervals for comparative purposes. This structured NRS approach ensured consistency and comparability across respondents’ subjective assessments.

## 3. Results

### 3.1. Demographics

**Figure 2:**
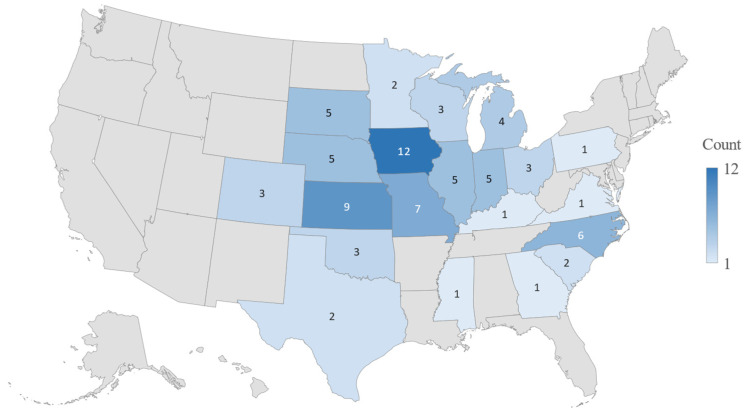
Geographic distribution of participating swine veterinarians in the United States.

A total of 46 responses were received, with a response rate of 23%. Of these, 27 responses were excluded from the final statistical analysis due to less than 80% completed or the participants stated that they were not directly responsible for swine medical treatment. The final statistical analysis included 19 surveys, resulting in an effective response rate of 9.5%. The 19 swine veterinarians included in the analysis had an average of 24.7 years of practice experience, and more than 78% of them had 15 or more years of swine medicine experience. On average, 482,736 pigs (including sows, growing and finishing pigs) were under direct or joint responsibility of each swine veterinarian at the time of the survey. As shown in Figure 2, the participants practiced across over 20 U.S. states, and 73% were located in Midwestern region, which is known for high concentration of pig populations.

### 3.2. Prioritization of Swine Diseases in Need of Better Vaccines

**Figure 3:**
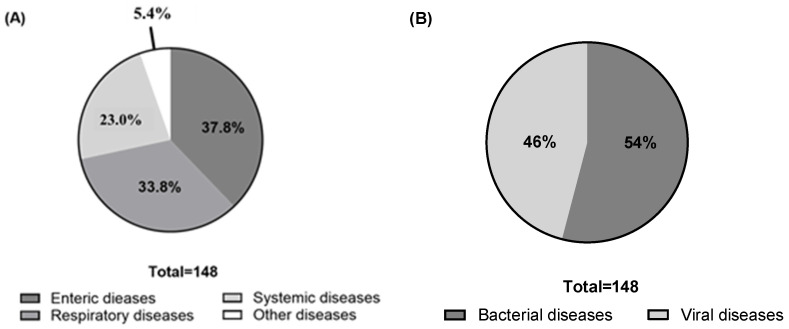
The categories of swine diseases in need of better vaccines. (**A**) By affected organ system. (**B**) By pathogen type.

Participants reported a total of 25 swine diseases that are in urgent need of better vaccines. As shown in Figure 3, these diseases were mentioned 148 times collectively. Based on affected organ system, enteric diseases were the most frequently reported (56 mentions, 37.8% of total), followed by respiratory diseases (50 mentions, 33.8%), systemic diseases (34 mentions, 23.0%), and other diseases (8 mentions, 5.4%). When categorized by pathogen type, viral diseases were mentioned 68 times, making up 45.3% of the total.

**Figure 4:**
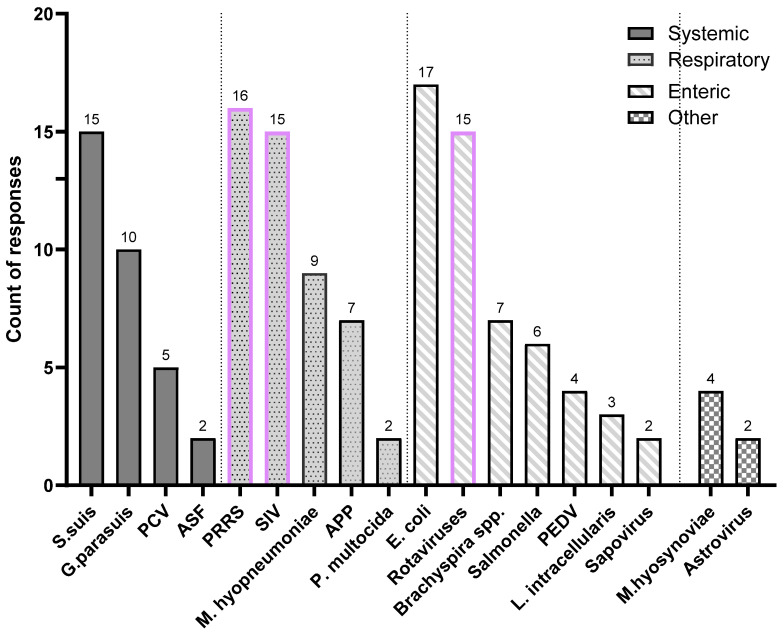
Top swine diseases in urgent need of improved vaccines. The purple boxes highlight the top three viral diseases in need of better vaccines.

Our findings on the prioritization of swine viral diseases are highlighted in Figure 4. The data reveal a strong consensus among veterinarians regarding which diseases urgently need improved vaccines. PRRS was the most frequently cited disease, with 84.2% of veterinarians (n = 16) identifying it as a top priority. Swine influenza (SIV) and rotaviral enteritis followed closely, each mentioned by 78.9% of the respondents (n = 15). Of all the viral diseases discussed, PRRS, SIV, and rotaviral enteritis were identified as the top three diseases most urgently in need of improved vaccine solutions.

### 3.3. Current Management Strategies for the Top Swine Viral Diseases

For the top three viral diseases, PRRS, SIV, and rotaviral enteritis, all participants reported using a combination of herd management, biosecurity measures, and vaccination as their primary disease management strategies. Additionally, over 25% of veterinarians noted using antimicrobials to address secondary bacterial complications that arise with these infections. Of the three, PRRS had the highest proportion of veterinarians relying on vaccines as a key management strategy (94%), as well as the highest proportion of using antimicrobial to control the secondary bacterial infections (56%). Notably, nearly all veterinarians were dissatisfied with the effectiveness of the current management strategies. On a 5-point scale, the average effectiveness score for each disease was approximately 2.5, placing them between “slightly effective” and “moderately effective” (Table 1).

### 3.4. Challenges of Current Vaccines for the Top Swine Viral Diseases

**Figure 5:**
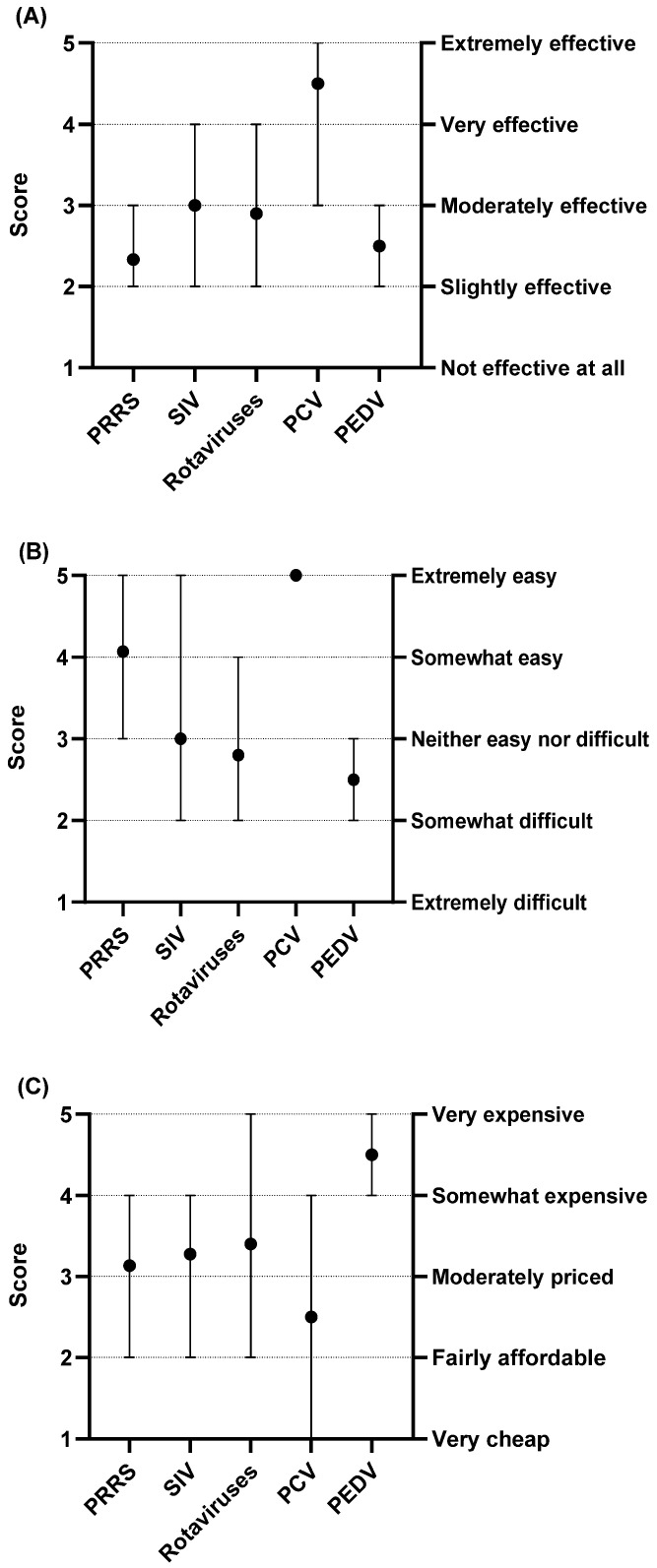
Rating current swine viral vaccines on effectiveness, availability, and cost (scores 1–5). (**A**) Effectiveness; (**B**) Availability; (**C**) Cost.

Vaccines are a critical tool in managing swine viral diseases, but our survey shows they have notable limitations. Figure 5 presents the average scores veterinarians assigned to the effectiveness, availability, and cost of current vaccines for PRRS, SIV, rotavirus, porcine circovirus (PCV), and porcine epidemic diarrhea virus (PEDV). For effectiveness, only the PCV vaccine performed strongly, with a mean score of 4.5 that falls between very effective and extremely effective. The other vaccines received substantially lower evaluations. PRRS had an average score of 2.3, while SIV and rotavirus averaged 3.0 and 2.9, respectively, and PEDV averaged 2.5. These four vaccines therefore clustered in the middle range of the scale (Figure 5A). For availability, while PRRS and PCV vaccines were generally rated as “somewhat easy” to acquire, those for SIV, rotavirus, and PEDV were perceived as less available, falling in the range of “neither easy nor difficult” to “somewhat difficult” (Figure 5B). For cost, PCV was the only vaccine considered relatively affordable, rated between “fairly affordable” and “moderately priced.” The other four vaccines were all rated on the more expensive side, between “moderately priced” and “very expensive” (Figure 5C). These scores highlight key areas for improvement. The following sections explore the specific limitations that contribute to these challenges in more detail.

#### 3.4.1. Specific Limitations That Contribute to Challenges on Effectiveness of Swine Viral Vaccines

When evaluating vaccine effectiveness, veterinarians rated the SIV (mean score = 3.0) and rotavirus (mean score = 2.9) vaccines as “moderately effective.” In contrast, the PRRS vaccine received a significantly lower average score of 2.3, placing it in the “slightly effective” category (Figure 5A). The most common challenge across all three diseases was a lack of cross-protection against new viral variants. This was cited by 88% of respondents for PRRS, 73% for SIV, and 47% for rotavirus. Beyond this shared issue, other challenges were disease-specific. For PRRS and SIV, the second most common challenge was the negative effect of maternal antibodies on vaccine efficacy, cited by 50% and 47% of veterinarians, respectively. For rotavirus, the second most common concerns were a short duration of protection and poor vaccine quality, both cited by 27% of respondents (Table 2).

#### 3.4.2. Specific Limitations That Contribute to Challenges on Cost and Availability of Swine Viral Vaccines

Veterinarians reported that PRRS, SIV, and rotavirus infections cause significant economic losses, estimated at 4.00, 2.00, and 0.89 dollars per head, respectively. Despite these substantial impacts, the mean cost ratings for the corresponding vaccines fell within the “moderately priced” range on the five-point scale (Figure 5C). To further contextualize these perceptions, veterinarians provided detailed information on both the per-dose cost and the estimated annual cost per head for each vaccine. For PRRS vaccines, the average cost per dose was 1.20 dollars, and the reported annual cost per head was 1.80 dollars, which corresponds to approximately 1.5 doses administered per pig each year. For SIV vaccines, the average cost per dose was 0.80 dollars, and the annual per-head cost was 1.80 dollars, indicating an average of about 2.25 doses per year. Rotavirus vaccines cost an average of 0.90 dollars per dose, with an annual per-head cost of 1.70 dollars, consistent with approximately 1.9 doses per year. Veterinarians feel that the vaccines’ limited effectiveness prevents them from providing a significant economic benefit. Since the vaccines do not adequately reduce disease incidence, producers are still forced to use costly antimicrobial treatments. This undermines the overall economic viability of vaccination and highlights the critical need for future improvements in vaccine effectiveness and quality.

When looking at the availability of swine viral vaccines, several key limitations were identified. (i) Limited vaccine administration method: All three vaccines for PRRS, SIV, and rotavirus were primarily administered through the intramuscular (IM) route. Only a small number of veterinarians reported using alternative methods, such as subcutaneous (SQ) for PRRS vaccine, subcutaneous (SQ) and intranasal (IN) for SIV vaccine, and both SQ and oral (PO) for rotavirus vaccine. (ii) Preference for vaccine types: Veterinarians showed a clear preference for commercial vaccines for PRRS. In contrast, autogenous vaccines were more frequently used for SIV and rotavirus (Table 3). This preference for autogenous vaccines for SIV and rotavirus appears to be a factor in their availability challenges.

For PRRS vaccine, it is rated as “somewhat easy” (mean score = 4.1). The main challenges were difficulties with administration and a shortage of storage. For SIV vaccine, it is rated as “neither easy nor difficult” (mean score = 3). The primary limitations were distribution delays and the high cost of customized autogenous vaccines. For rotavirus vaccine, it is rated as “somewhat difficult” (mean score = 2.8). Similar to SIV, its availability was affected by distribution delays and the high cost of customized autogenous vaccines (Table 4). These findings suggest that the higher use of autogenous vaccines for SIV and rotavirus is directly linked to the reported challenges of distribution delays and cost.

#### 3.4.3. Specific Limitations That Contribute to Challenges on Safety of Vaccines

While participants reported no safety concerns regarding rotavirus vaccines, several issues were raised concerning the safety of vaccines for PRRS and SIV. The primary safety concerns for PRRS vaccines were related to their potential impact on herd productivity and a risk of reverting to virulence. 56% of participants expressed concern that the vaccine could cause a temporary reduction in the productivity of their swine herds. 56% of participants, who feared that the weakened live virus in the vaccine could mutate back into a more harmful, disease-causing form. These concerns reflect practitioners’ field experience rather than direct evidence from this study, but they help explain why some veterinarians remain cautious when recommending PRRS MLVs. For SIV vaccines, the main safety concern was tied to adverse physical reactions at the injection site. This was the most commonly reported concern, with 33% of participants noting issues such as swelling, redness, or abscesses at the injection site (Table 5).

### 3.5. Future Perspectives and Opportunities for Improved Swine Viral Vaccines

**Figure 6:**
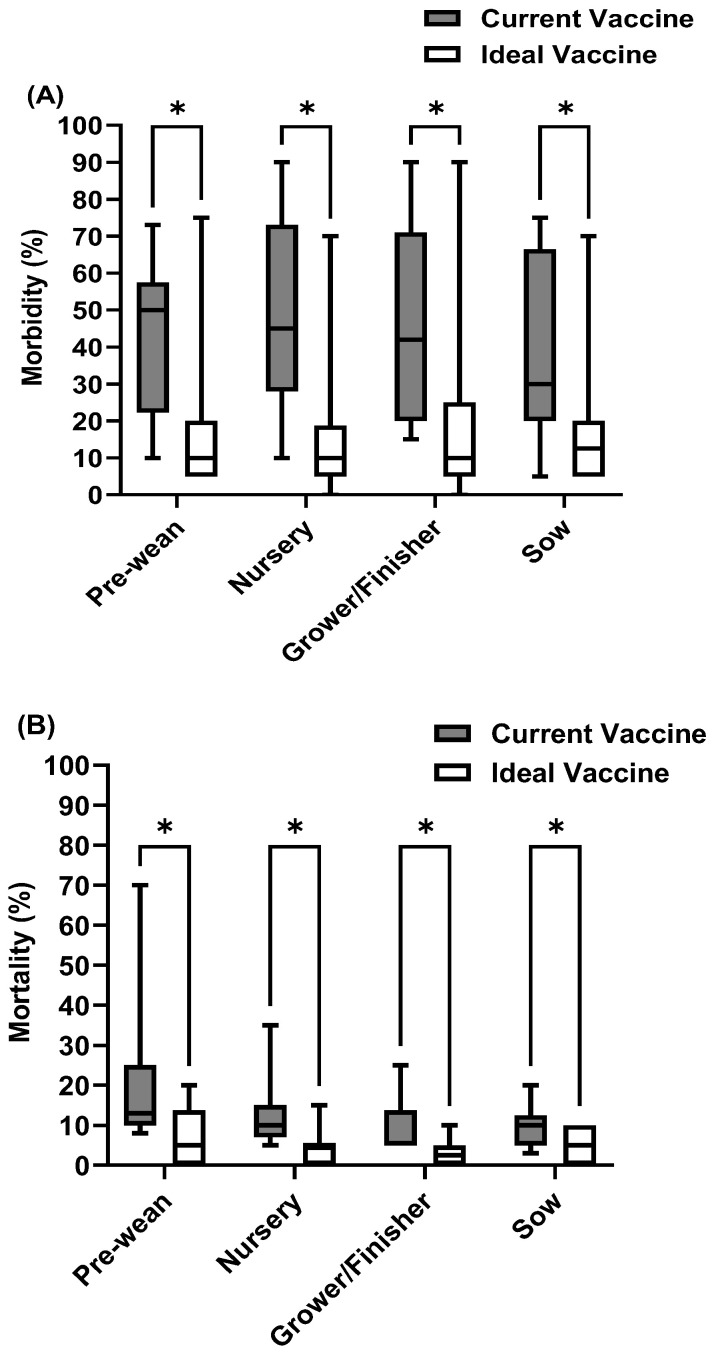

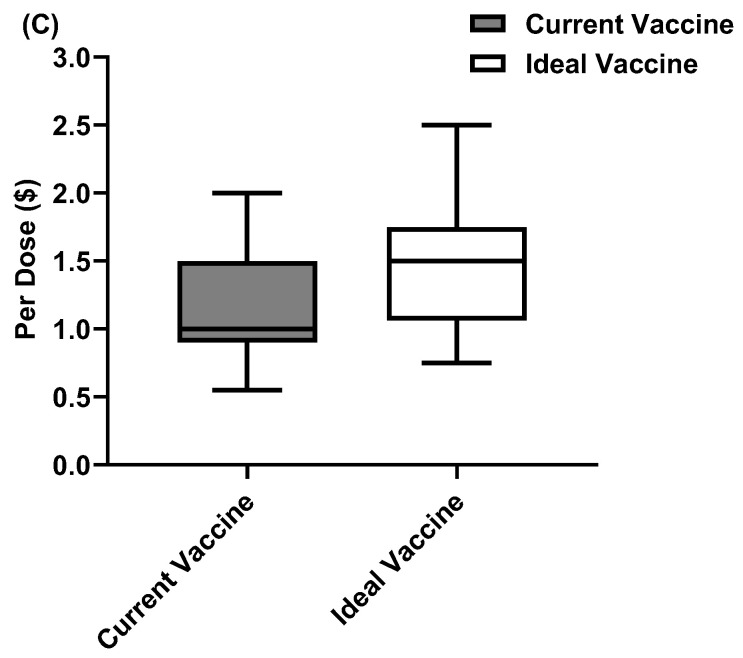
PRRS current vaccine compared with ideal vaccine in terms of (**A**) Morbidity, (**B**) Mortality, and (**C**) Cost. “*” indicates “significant difference (*p* < 0.05)”.

**Figure 7:**
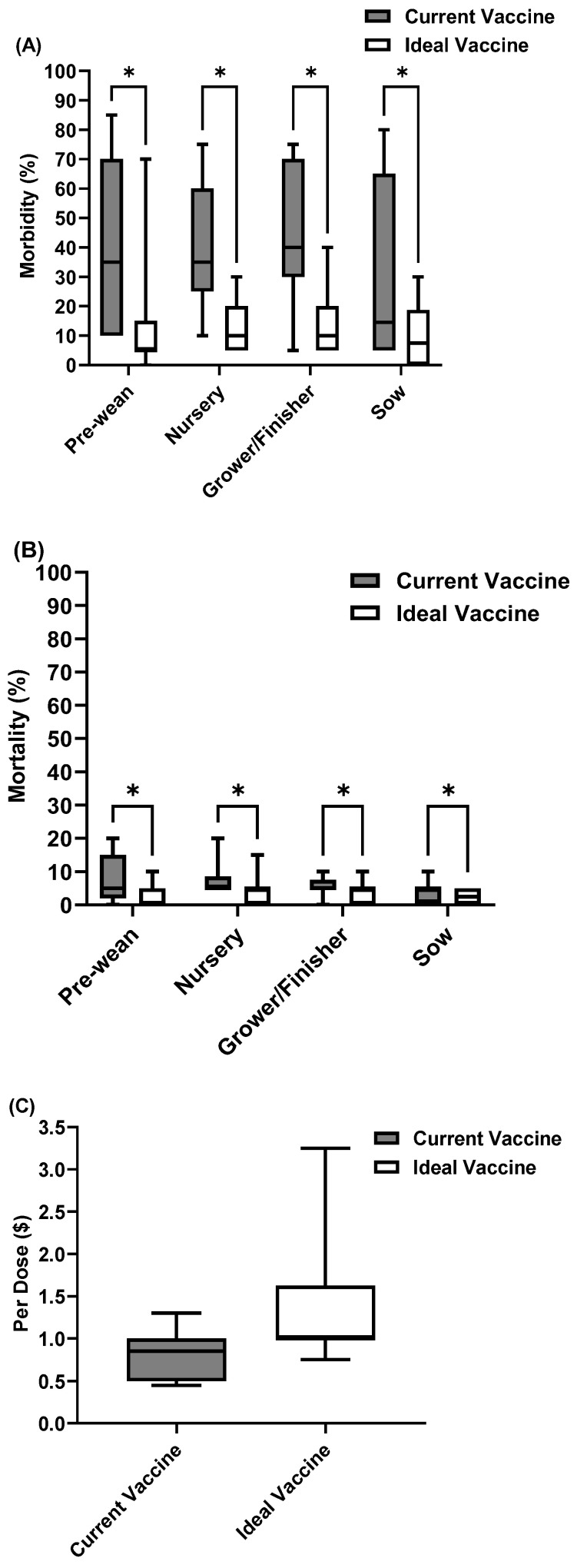
SIV current vaccine compared with ideal vaccine in terms of (**A**) Morbidity, (**B**) Mortality, and (**C**) Cost. “*” indicates “significant difference (*p* < 0.05)”.

**Figure 8:**
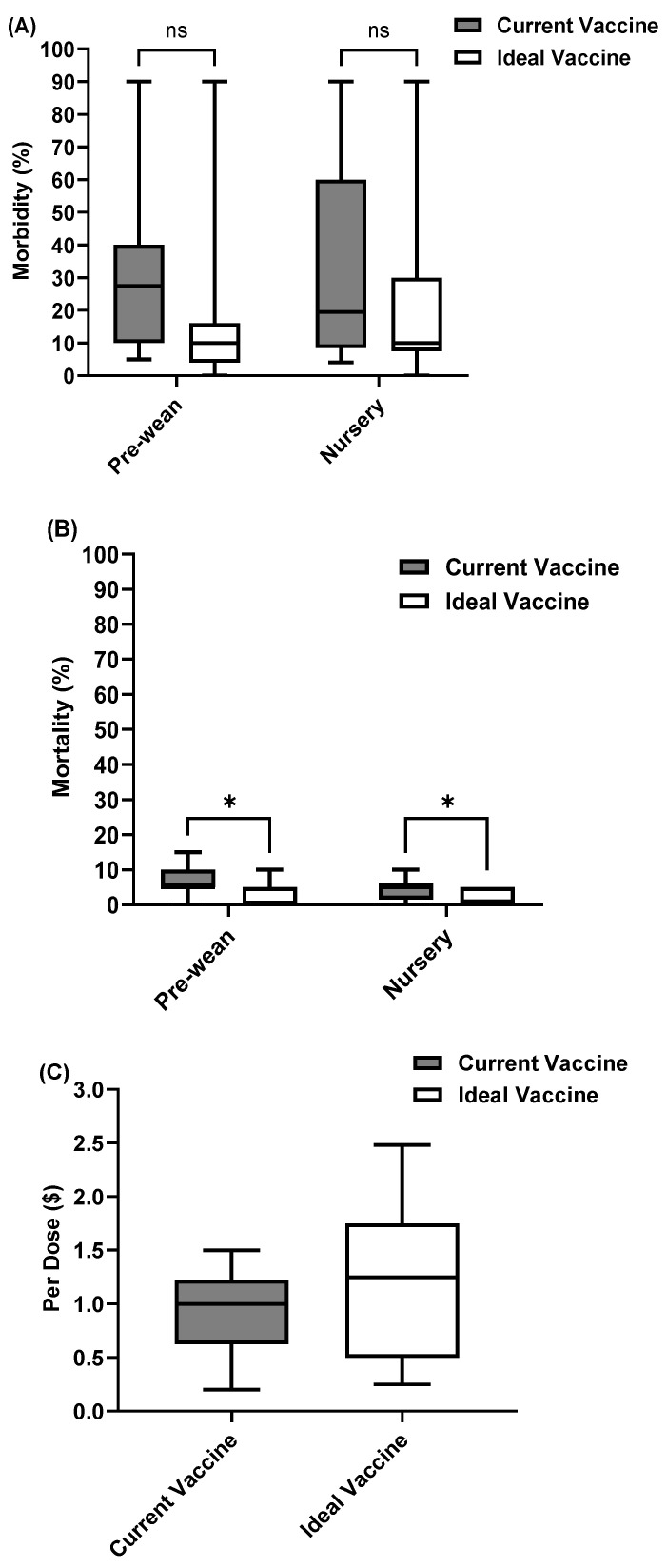
Rotavirus current vaccine compared with ideal vaccine in terms of (**A**) Morbidity, (**B**) Mortality, and (**C**) Cost. “*” indicates “significant difference (*p* < 0.05)”. “ns” indicates “no significant difference”.

A significant issue shared across all three diseases is a lack of cross-protection. This means the current vaccines do not provide broad immunity against different strains or types of viruses. As a result, they are not as effective as they could be in reducing morbidity and mortality rates (Figure 6, Figure 7 and Figure 8). To address this, we asked veterinarians to estimate the potential decrease in disease incidence if the main vaccine challenges were overcome. Their responses highlighted a strong belief in the value of improved vaccines and a clear willingness to invest more, despite current economic pressures. Specifically, they would pay 28% more for a better PRRS vaccine, 66.3% more for an improved SIV vaccine, and 35.6% more for a superior rotavirus vaccine. This indicates a strong market demand for more effective and safer swine viral vaccines, even at a higher cost, due to the expected benefits for disease control and overall herd health.

## 4. Discussion

### 4.1. Top Swine Viral Diseases and Their Concerns

This study investigated U.S. swine veterinarians’ perspectives on using viral vaccines to reduce or replace antimicrobial use. The survey identified PRRS, SIV, and rotavirus as the top three swine viral diseases most in need of improved vaccines. This finding aligns with recent surveillance from the Swine Health Information Center, which has reported the rapid spread of PRRS virus lineage 1C.5 and an increase in Influenza A virus cases, especially in wean-to-finish facilities [7]. Rotavirus also remains a leading cause of pre-weaning mortality in Midwest U.S. herds [8]. These viruses contribute to increased antimicrobial use by suppressing the immune system and damaging physical barriers; for example, PRRS targets immune cells [9], while SIV and rotavirus compromise respiratory and intestinal barriers, respectively [10]. These weakened host defenses leave pigs vulnerable to secondary bacterial infections. Our survey respondents confirmed they use more antimicrobials to address these complications, particularly in PRRS-positive herds. This observation is supported by previous studies that noted a 379% increase in antimicrobial use in nursery pigs following PRRS outbreaks [6].

Veterinarians also expressed substantial concern regarding the limited cross-protection provided by current commercial vaccines, particularly against emerging variants. Although many respondents continue to use PRRS modified-live virus (MLV) vaccines, a considerable number rely on autogenous vaccines for SIV and rotavirus (Table 3), highlighting gaps in the performance of available products. Each vaccine type was associated with specific limitations. For PRRS, concerns centered on temporary reductions in productivity, safety issues, and the persistence of vaccine virus viremia. Respondents noted that prolonged viremia can complicate diagnostic interpretation and may increase the risk of recombination with wild-type strains [11,12,13]. For SIV, respondents reported that most commercial vaccines available to them were whole-inactivated virus (WIV) formulations administered to sows, a strategy primarily dependent on passive immunity. However, maternally derived antibodies can interfere with vaccine-induced protection; while homologous MDA offers some protection, heterologous MDA may not only fail to prevent disease but can exacerbate clinical signs following post-weaning exposure [14,15]. For rotavirus, most commercial vaccines target rotavirus A (RVA), even though rotavirus C (RVC) has emerged as a major cause of neonatal diarrhea [16]. A nationwide study reported RVC in 76.1% of pre-weaning piglets with clinical diarrhea, yet no effective commercial vaccine is available due to the lack of a suitable cell culture system for its development [17,18]. These limitations highlight the urgent need for vaccines that induce broader cross-protective immunity.

### 4.2. Promising Strategies for Vaccine Improvement of the Top Swine Viral Diseases

For PRRS, switching from intramuscular (IM) to intradermal (ID) injection could significantly improve PRRS MLV vaccine safety. ID administration can shorten viremia duration, reduce viral shedding, and limit tissue damage, which are key safety concerns with MLV vaccines [19,20,21,22]. This method also requires a lower dose and causes less stress and tissue damage in pigs, suggesting reduced inflammatory responses and improved post-vaccination recovery [23]. However, further research is needed to evaluate its effectiveness in field conditions and assess its practicality for large-scale commercial swine production.

For SIV, current vaccines are mostly WIV formulations, which are susceptible to MDA interference and have been linked to vaccine-associated enhanced respiratory disease (VAERD) [14]. As an alternative, adenovirus-vectored vaccines can induce strong, long-lasting humoral and cellular immune responses [24]. Crucially, these vaccines can bypass MDA interference and reduce viral shedding, addressing major limitations of WIV vaccines [25]. Given that MDA interference was identified as a major cause of SIV vaccine failure in our study, these findings highlight the potential of adenovirus-vectored platforms as a promising strategy for improving SIV control.

For rotavirus, since piglets are highly susceptible and exposed to rotavirus immediately after birth, a primary strategy is to enhance maternal immunity. While natural planned exposure (NPE) is currently the main method to stimulate passive lactogenic immunity in sows to protect piglets from RVC infection, it carries risks like shedding of RVC and introducing unintended pathogens [26,27]. Prescription platform vaccines, like those developed by Medgene Labs and SEQUIVITY from Merck Animal Health, offer a promising alternative. These platforms use a standardized production system with a single vector or expression backbone. By inserting specific genes of interest (GOI) coding for immunogenic proteins of RVC into this system, they can rapidly produce non-replicating vaccines tailored to the prevalent strains in a particular herd or region. This technology can significantly improve immunization efficacy. The current production timeline of 12–16 weeks remains a limitation, but optimizing expression systems could reduce this time and make the approach more feasible for rapid response.

### 4.3. Barriers and the Role of Veterinarians

The top three viral diseases create a significant economic burden, with losses estimated at $4.10 per pig for PRRS, $2.10 for SIV, and $0.90 for rotavirus. While vaccination is a key tool for mitigating these losses, concerns about cost-effectiveness persist. Our study found the average per-dose cost for commercial PRRS, SIV, and rotavirus vaccines to be $1.16, $0.83, and $0.90, respectively. Despite perceiving these prices as somewhat expensive, veterinarians showed a strong willingness to pay more for improved vaccines, with acceptable price increases of 28% for PRRS ($1.48), 66.3% for SIV ($1.38), and 35.6% for rotavirus ($1.22). This finding demonstrates a clear understanding that the long-term economic benefits of effective disease control outweigh the initial vaccine cost. However, cost remains a significant barrier, especially for small to medium-sized operations with tighter margins. For example, the Morrison Swine Health Monitoring Project found that only about 30% of surveyed U.S. sow farms had adopted PRRS vaccination [28]. Adoption rates could be improved through cost-sharing mechanisms, government subsidies, or more effective vaccines [29]. Many producers still see antibiotics as a more cost-effective management strategy, often underestimating the long-term financial benefits of vaccination, such as reduced antimicrobial use and improved herd health.

Swine veterinarians play a central role in the veterinarian–client–patient relationship (VCPR) and are at the forefront of disease prevention, control, and biosecurity in swine production [30]. Maintaining herd health is not a static process; rather, it requires continuous evaluation and adjustment of management strategies. As trusted advisors to producers, veterinarians apply their expertise to provide critical guidance on herd health management, including vaccination protocols and antimicrobial use strategies [31,32]. However, the decision-making process regarding disease management is complex and influenced by various factors [33]. Understanding how veterinarians weigh the use of vaccines versus antimicrobials is crucial to identifying the factors that shape their recommendations. These considerations underscore the importance of veterinarians as the key focus of this study.

Despite their significant role, there is a notable lack of research on veterinarians’ perspectives regarding the effectiveness, safety, and economic feasibility of viral vaccines as alternatives to antimicrobials in swine production. This gap limits our understanding of how veterinarians perceive vaccine limitations and their practical application in real-world production settings. By addressing this gap, our study provides valuable insights from their practical experiences and concerns, which can contribute to future vaccine development and policy decisions.

### 4.4. Limitations of the Study and Future Directions

This study has potential limitations that should be considered. The primary limitation is the small sample size; of the 200 veterinarians invited to participate, only 19 completed more than 80% of the survey. Another limitation of this study is the length of the questionnaire. The extensive length of the questionnaire, which covered 13 different pathogens and took an average of over 30 min to complete, likely contributed to the low response rate and incomplete responses. This factor should be considered when interpreting the representativeness of the survey findings.

Although only five interviews were conducted, the participants were experienced swine veterinarians with an average of more than 20 years of practice and worked with medium-to-large-scale commercial herds. The main themes related to vaccine limitations, economic considerations, and challenges in vaccine adoption began to recur across interviews, indicating that substantial saturation was reached. However, the small number of interviews still limits the breadth of perspectives captured.

Future research should build on this foundation with more targeted investigations. Integrating production data from swine farms would complement veterinarians’ perspectives and reduce reliance on recall-based responses. Combining these insights with multi-year diagnostic or surveillance data would also provide valuable epidemiological context. Longitudinal information on the circulation of PRRSV, SIV, and rotavirus at the farm or regional level could help strengthen vaccine development strategies and support more informed disease management decisions. Although such analyses were beyond the scope of the present perception-based survey, they could meaningfully extend the findings presented here. Studies with larger sample sizes and field level assessments will further improve our understanding of vaccine limitations and inform more precise development strategies.

## 5. Conclusions

This study offers key insights from U.S. swine veterinarians on the challenges and opportunities of using viral vaccines as an alternative to antimicrobials or to reduce antimicrobial use. Our findings highlight an urgent need for improved vaccines for PRRS, SIV, and rotavirus, which cause significant economic losses and increase the need for antimicrobials. Veterinarians identified major challenges with current vaccines, including limited cross-protection, safety concerns, and issues with cost and availability. However, despite these limitations, they expressed strong support for using vaccines to reduce antimicrobial reliance. Addressing these gaps will require future research focused on targeted vaccine development and a better understanding of the factors that influence veterinarians’ decision-making. Ultimately, these efforts will support more effective disease control, reduce antimicrobial use, and protect both animal and public health.

## Figures and Tables

**Table 1:** Summary of current management strategies and effectiveness scores for the top three swine viral diseases.

Disease	Current Management Strategies	Percentage	Score
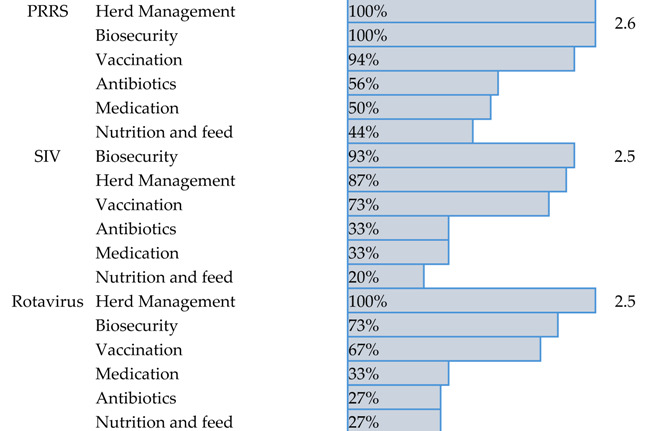			

**Table 2:** Specific limitations contributing to vaccine ineffectiveness for the top three swine viral diseases.

Disease	Effective Limitations	Percentage
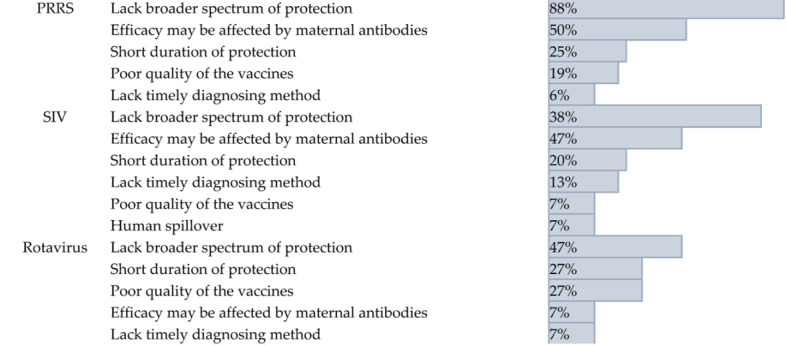		

**Table 3:** Summary of respondents’ answers on the type of swine viral vaccine currently used.

Group	Commercial Vaccines	Autogenous Vaccines
PRRS	100%	37.5%
SIV	46.7%	66.7%
Rotavirus	26.7%	60%

**Table 4:** Summary of respondents’ answers on the availability limitations of current swine viral vaccines.

Disease	Availability Limitations	Percentage
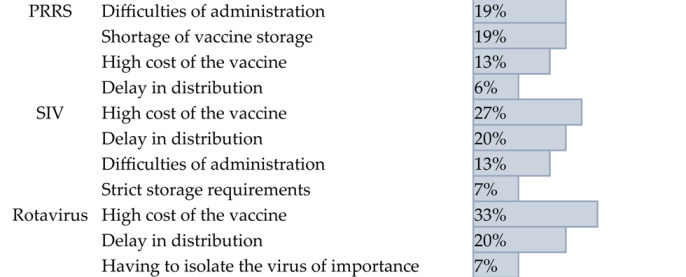		

**Table 5:** Summary of respondents’ answers on the adverse effects of current swine viral vaccines.

Disease	Adverse Effects	Percentage
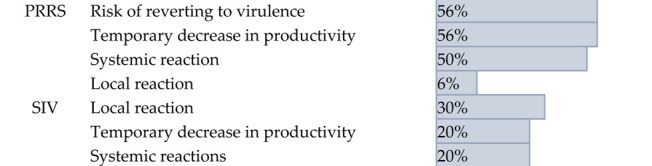		

## Data Availability

The datasets used and/or analyzed during the current study are available from the corresponding author upon reasonable request.
